# Knee and hip agonist-antagonist relationship in male under-19 soccer players

**DOI:** 10.1371/journal.pone.0266881

**Published:** 2022-04-15

**Authors:** Filipe Rosa, Hugo Sarmento, João Pedro Duarte, Joel Barrera, Francisco Loureiro, Vasco Vaz, Nestor Saavedra, António José Figueiredo

**Affiliations:** 1 University of Coimbra, Research Unit for Sport and Physical Activity (CIDAF), Faculty of Sport Sciences and Physical Education, Coimbra, Portugal; 2 Research Group in Sports Science and Physical Activity, Faculty of Health Sciences, Sports Science Program, University of Applied and Environmental Sciences, Bogota, Colombia; Instituto Politecnico de Viana do Castelo, PORTUGAL

## Abstract

The purpose of this study was to evaluate the strength of the knee flexors and extensors and hip abductor and adductor muscles in young soccer players. Twenty-three male under-19 soccer players participated in this study (age: 17.7 ± 0.2 years; height: 173.0 ± 1.1 cm; body mass: 66.1 ± 1.3 kg). Body composition was measured using a bioelectrical impedance (InBody770), and the dynamometry was performed by an isokinetic dynamometer (Biodex System 3) for knee flexion and extension, and by an isometric dynamometer (Smart Groin Trainer), for hip adduction and abduction. Comparisons were made between dominant members (D) vs. non-dominant members (ND) and adductors vs. abductors (ADD:ABD) using the Wilcoxon test. There were statistically significant differences in the peak torques between the dominant and non-dominant members in the flexion function (Z = −4.198, p < 0.01) and in the extension function (Z = −4.197, p < 0.01) of the knee in concentric muscular action, and the flexion (Z = −4.198, p < 0.01) and in the extension (Z = −4.198, p < 0.01) of the knee in eccentric muscular action. No statistically significant differences were obtained in the conventional ratio (Z = −0.456, p = 0.648) nor the functional ratio (Z = −0.335, p = 0.738) between D and ND members. There were no statistically significant differences between adductors and abductors at the moment of strength for absolute values (N). The reference absolute and normalized to the weight values and the ADD:ABD can be used as a guideline for classifying players in screening and comparison in return tests to sports practice after an injury.

## Introduction

Soccer as increased match-play physical demands due to short between-match recovery periods and high neuromuscular demands (e.g., a larger number of high-intensity running actions, acceleration/deceleration requirements, changes of direction, and jumping/landing tasks) [1]. Muscle strength is crucial for performance and injury prevention. Soccer has a high injury rate and percentage, where the three most commonly injured body segments/joints, for male players, are the ankle (20%), the thigh (17%), the knee (15%), and the hip/groin (6%) [2–4].

Strength differences between legs (bilateral strength asymmetries) and between muscle groups (agonist-antagonist relationship) have been reported in athletes [5]. Soccer-specific actions provide the unilateral overuse of lower limbs in almost all passing/receiving, kicking, and cutting skills and soccer players are more effective in unilateral strength training exercises than athletes who frequently perform bilateral activities [6, 7]. Also, strength asymmetry seems desirable for improving performance in soccer-related abilities, once was shown to account for better kicking performance with the dominant limb (D) [8]. Previous literature indicated 56% of the pre-season period players are at risk of muscle strength imbalances for knee flexors (KF) or extensors (KE) [9] concerning the knee function, professional soccer level practice, and competition as critical factors for asymmetries [7]. Risk factors associated with the development of groin injuries are classified as modifiable and non-modifiable [10]. The modifiable factors are body mass, decreased adductor muscle strength, decreased hip abduction range, total hip rotation range, and adductor:abductor strength ratio (ADD:ABD) [10–12].

Injury’s burden (muscle/ tendon injuries to the hamstrings and groin, ligament sprains, and joint injuries to the knee and ankle) are more likely to impact team performance negatively [13]. Screening is essential to obtain a comprehensive assessment of the athlete at the baseline level and to identify risk factors for injury, health conditions, and musculoskeletal conditions that require follow-up or intervention [14]. For example, hip strength is a risk factor; it should be included in the pre-season screening or more regularly during the season since some individuals lose strength two weeks before the onset of groin injury [11, 15]. In addition, screening the quadriceps and hamstrings peak torque allows evaluating inter-limb or anterior-posterior asymmetry in strength, which is used to monitor muscle strength asymmetries that are strongly correlated with high risk for hamstrings strain injury [16]. Indeed, several studies have investigated the hamstrings-to-quadriceps peak torque ratio [17–19] or the inter-limb asymmetry in hamstrings peak torque [4–6], and also the ADD:ABD [10, 11] and their relationship with injury risk in soccer players. Since soccer requires movements in different descriptive planes, it makes sense to evaluate the musculature and strength production, considering more than one of these plans to understand better each muscle group’s contribution and role while making a technical gesture. Thus, the purpose of this study was to evaluate the strength of the KF and KE and hip abductor and adductor muscles in young soccer players, b) determination of conventional and functional ratios of the KE and KF muscles and hip adductors and abductors; b) determination of bilateral differences between the D and non-dominant limb (ND). Based on the literature review, it is hypothesized that young soccer players present bilateral strength asymmetries and muscle imbalances of the knee joint muscles influenced by practical experience and morphological differences.

## Methods

### Participants

Twenty-three male U-19 soccer players (age: 17.7 ± 0.2 years; height: 173.0 ± 1.1 cm; body mass: 66.1 ± 1.3 kg) took part in this prospective study. The design’s power and sample size were calculated using the G-Power software (University of Dusseldorf, Dusseldorf, Germany) with the following parameters: Wilcoxon signed-rank test (matched pairs); t tests; a priori. Input parameters: one tail, effect size = 0.5 α err prob = 0.05, power = 0.8. Output parameters: noncentrality = 2.6, df = 25.7, total sample size = 23 players, actual power = 0.81. Fifteen participants were right-leg dominant, and eight were left-leg dominant. Chronological age was determined to the nearest 0.1 year by subtracting birth date from the date of first testing measurement. Training experience was obtained by questionnaire and confirmed in Portuguese Federation records. The study follows ethical standards for Sports Medicine with human samples [20]. All participants were informed of the purpose and content of the study and provided written informed consent prior to participation. In the case of underage participants, parents or legal guardians were also thoroughly informed and signed the consent on their behalf. The study was approved by the Scientific Council and the Ethics Committee of the University of Coimbra (CE/FCDEF-UC/00692021) and conducted according to the Declaration of Helsinki. As inclusion criteria: a) to have more than ten years of training experience and federated competing; b) to belong to a structured and certified soccer academy. The exclusion criteria were: a) a history of knee, hip, and groin orthopedic problems within the previous twelve months to the onset of the investigation (no players excluded); b) inability to undertake testing due to other injuries (two players excluded).

### Study protocol

All tests were carried out at the end of the pre-season, during the same period of the day (five consecutive mornings), in similar atmospheric and climatic conditions, in the same equipment as the integrated Biokinetics laboratory, following the same warming-up routine, sequence of protocols (questionnaires, anthropometry, body composition, and strength tests (knee and hip joints), and respect for the same rest times, always under the guidance of the same observers.

### Anthropometry and total body composition

Height was measured to the nearest 0.1 cm using a Harpenden stadiometer (model 98.603, Holtain Ltd., Crosswell, GB). Body mass was obtained using a scale SECA (model 770, Hanover, MD, USA) with 0.1 kg reduction. To avoid the influence of food intake or exercise on bioelectrical impedance measurements, all subjects had no food or liquid intake before at least 12 hours and no diuretic medication on the previous week. In addition, body composition (fat mass [kg and %]) was measured using a valid multi-frequency segmental bioelectrical impedance analyzer (InBody770, Biospace, Seoul, Korea). When assessing body fat-mass, in 50 males athletes, this instrument delivered comparable measurements, and the degree of agreement was excellent (r2 = 0.955; CV% = 0.15; ICC = 0.960) compared with the reference method (dual X-ray absorptiometry) [21].

### Appendicular thigh volume

The thigh volume (TV), estimated by anthropometry, of both legs was determined from three circumferences and two partial lengths, based on the procedures proposed by Jones and Pearson [22]. The thigh volume is fractioned into two portions, like two truncated cones. The first truncated cone was determined from circumferences measured at the most proximal gluteal furrow and one-third of the subischial height up from the tibial–femoral joint space. The second truncated cone was similarly determined considering circumferences at the one-third of the subischial height up from the tibial-femoral joint space and at the transversal plan above the patella. In Briefly, volume was estimated from an equation derived from proximal (A1) and distal (A2) area sections that composed the truncated cones, and L is the length between the two transverse plans:

$$TV={\left([A1+A2+(A1\times A2)\right]}^{0.5})\times \frac{1}{3}\times L$$ (1)

The areas A1 and A2 were derived from leg circumferences:

$$A=\frac{C^{2}}{4\times \pi }$$ (2)

### Isokinetic knee strength test

Both lower limbs were assessed in a validated Biodex System 3 dynamometer (Shirley, NY, USA). Before the assessment session, dynamometer calibration and positioning were performed, following the manufacturer’s instructions (Biodex Medical Systems, Inc., 2000) and previous work [19]. The global range of movement was set at 85°, with from 5° to 90° of flexion as follows: soccer players were asked to perform a voluntary maximal knee extension, and the 0° was settled; afterward, the initial 5 degrees of the flexion were completed and the dynamometer blocked. The reduction of the flexion’s initial angles was made to allow the athlete to exert at least 10% of the assigned torque limit [19]. Individual calibration of gravity was corrected before each test at 30 degrees of knee flexion [23]. To familiarize the soccer players and to attenuate the learning curve, before starting the test, and as recommended, specific 3-trial repetitions at the same speed and action were performed [18]. During the test, participants were instructed to keep their arms crossed with the hands-on opposite shoulder [24]. The computer screen linked to the dynamometer gave consistent real-time visual feedback [25]. The warm-up protocol consisted of 5-minutes peddling in a cycle ergometer (814E Monark, Varberg, Sweden) with a resistance braking force corresponding to 2% of the participant’s body mass, cycling between 50 and 60 rpm [24]. Reciprocal concentric (con) and eccentric (ecc) muscular actions were tested considering 5 repetitions for each movement at 60°/s (1.05rad/s). A 60-second interval was settled between the 3-repetition familiarization and the test [24]. Outputs were analyzed with the *Acqknowledge* software version 4.1 (Biopac Systems, Inc., Goleta, CA, USA) according to previous work [19]. The PT value of the best five repetitions was retained for analysis (best curve performed by KE and KF in both the con and ecc muscular actions: KEcon, KEecc, KFcon, KFecc), and composite ratios. Eq 3 corresponding to the functional ratio [17].


KFecc/KEcon (3)

Dominant (D) to Non-Dominant (ND) bilateral difference was determined using the Eq 4 [26] and according previous research [27].


((D−ND)/D)×100 (4)

### Adductor squeeze and abductor strength test

Each participant laid supine on a bench and, as explained elsewhere [28]. Strength was quantified using a handheld dynamometer (Smart Groin Trainer, Neuro Excellence, Portugal), reliable, and low error values [29]. Hips were positioned in a 45° flexion with knees flexed to 90° and hips in neutral rotation. The dynamometer was placed between the knees; specifically, it was located at the most prominent point of the medial femoral condyles. Players were instructed to squeeze (for adduction) the cuff as hard as possible for three maximum contractions and held for five seconds with three minutes of passive recovery between contractions. The inverse was required for abduction. The maximal pressure (squeeze) value displayed on the dynamometer dial was recorded during each of the three test trials and converted for Newtons. The best of the three trials was used for analysis.

### Statistical analysis

Descriptive statistics consisted of a measure of central tendency, mean [standard error (SE) of the respective and 95% confidence interval (CI)], and standard deviation as a measure of dispersion. Normality was checked with the Shapiro-Wilk test, and when the premises were violated, a logarithmic transformation was performed to reduce the non-standardized error. Then, comparisons were made between members D vs. ND and Adductors vs. Abductors using the Wilcoxon test (nonparametric). Statistical significance was established at p <0.05. All analyzes were performed using the statistical software Statistical Package for the Social Sciences, version 26 (SPSS Inc., IBM Company, NY). The figure prepared in version 9.1 of the GraphPad Prism software (GraphPad Software, Inc.; La Jolla, CA, USA).

## Results

Table 1 summarizes descriptive statistics for chronological age, training experience, anthropometry, body composition, and thigh volumes. Body size, body composition, and appendicular thigh volumes parameters were normally distributed. From the whole sample, no soccer player suffers groin or knee injuries during the competitive season.

**Table 1:** Descriptive statistics and normality test for the total sample considering crhonovariables and anthropometry (n = 23).

variables	units	mean			SD	Shapiro-Wilk	
		value	SE	95% CI		value	*p*
Chronological age	years	17.7	0.8	(17.3 to 18.0)	0.2	0.897	0.022
Training experience	years	11.9	1.7	(11.1 to 12.5)	0.4	0.846	<0.01
Stature	cm	173.0	5.4	(171.0 to 175.4)	1.1	0.941	0.187
Body mass	kg	66.1	6.2	(63.4 to 68.6)	1.3	0.967	0.615
Fat mass	kg	9.0	2.2	(8.1 to 10.0)	0.5	0.889	0.015
Fat mass	%	13.5	2.6	(12.5 to 14.6)	0.5	0.920	0.067
Left appendicular thigh volume	L	4.7	0.6	(4.4 to 4.9)	0.1	0.954	0.350
Right appendicular thigh volume	L	4.6	0.6	(4.3 to 4.9)	0.1	0.982	0.942

As expected, KE values are higher than the KF for all variables. The same for muscular actions, where reactive mode (ecc) presented superior values than voluntary evaluation (con) (Table 2). Considering the hip joint absolute and relative values are similar for adductors and abductors.

**Table 2:** Descriptive statistics and normality test for the total sample considering strength parameters (n = 23).

variables	units	mean			SD	Shapiro-Wilk	
		value	SE	95% CI		value	*p*
Dominant PT KEcon	N.m	195.5	36.7	(181.1 to 211.4)	7.7	0.983	0.950
Non-Dominant PT KEcon	N.m	173.7	336	(160.1 to 187.8)	7.0	0.940	0.177
Dominant PT KFcon	N.m	112.1	29.2	(98.9 to 123.5)	6.1	0.933	0.125
Non-Dominant PT KFcon	N.m	98.3	30.8	(84.3 to 110.0)	6.4	0.896	0.021
Dominant conventional ratio		0.58	0.13	(0.52 to 0.62)	0.03	0.890	0.016
Non-Dominant conventional ratio		0.57	0.15	(0.50 to 0.62)	0.03	0.893	0.018
Dominant PT KEecc	N.m	239.4	55.8	(218.1 to 263.1)	11.6	0.977	0.859
Non-Dominant PT KEecc	N.m	211.6	51.4	(193.1 to 233.7)	10.7	0.954	0.355
Dominant PT KFecc	N.m	148.3	32.4	(134.5 to 162.2)	6.8	0.959	0.450
Non-Dominant PT KFecc	N.m	127.6	27.4	(116.5 to 139.2)	5.7	0.969	0.663
Dominant functional ratio		0.77	0.16	(0.71 to 0.83)	0.03	0.885	0.012
Non-Dominant functional ratio		0.74	0.15	(0.69 to 0.81)	0.03	0.923	0.077
T Adductors	N	468.2	69.9	(435.3 to 501.9)	18.1	0.934	0.313
T Adductors	N/kg	7.1	1.1	(6.6 to 7.7)	0.3	0.793	0.003
T Abductors	N	473.2	80.5	(429.9 to 412.4)	20.8	0.969	0.844
T Abductors	N/kg	7.1	1.0	(6.7 to 7.7)	0.3	0.926	0.239

PT (Peak Torque); T (Torque); KE (Knee Extensors); KF (Knee Flexors); con (concentric); ecc (eccentric); SE (Standard Error); CI (Confidence Interval); SD (Standard Deviation).

Table 3 presents the comparative statistics between lower-limbs considering the KE and KF muscles. There are bilateral differences (p<0.05) between D and ND limbs. Ecc muscular actions revealed higher absolute differences than con muscular actions (i.e., 27.8 N.m for KE and 28.3 for KF). Regarding ratios, there are no statistically significant differences between limb D and ND in the conventional ratio (Z = –0.456, p = 0.648) nor in the functional ratio (Z = –0.335, p = 0.738).

**Table 3:** Comparative statistics for knee extensors, knee flexors and ratios considering dominant and non-dominant lower-limbs.

variables	Dominant		Non-Dominant		Wilcoxon	
	mean	SD	mean	SD	Z	p
PT KEcon	195.5	7.7	173.7	7.0	–4.197	<0.01
PT KFcon	112.1	6.1	98.3	6.4	–4.198	<0.01
Conventional ratio	0.58	0.03	0.57	0.03	–0.456	0.648
PT KEecc	239.4	11.6	211.6	10.7	–4.198	<0.01
PT KFecc	200.6	6.8	172.3	5.7	–4.198	<0.01
Functional ratio	0.77	0.03	0.74	0.03	–0.335	0.738

PT (Peak Torque); KE (Knee Extensors); KF (Knee Flexors); con (concentric); ecc (eccentric); SD (Standard Deviation).

**Fig 1:**
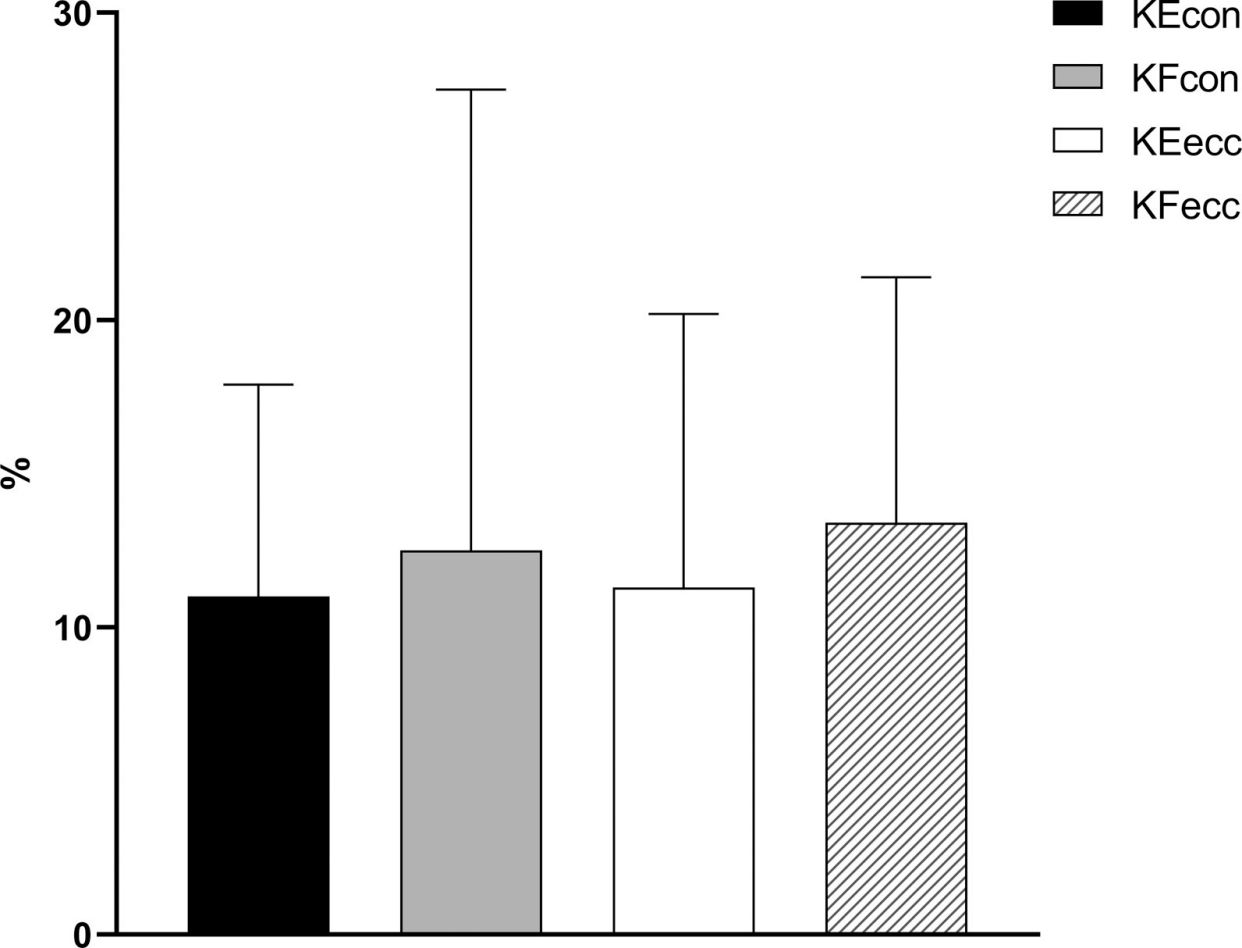
Dominant vs. non-dominant bilateral differences (%) for the knee joint. KE (Knee Extensors); KF (Knee Flexors); con (concentric); ecc (eccentric).

Bilateral differences (%) are examined in Fig 1. For KE, bilateral differences percentages are similar (i.e., 11%) in both muscular actions. However, the KF muscle group has higher (12.5 and 13.4) differences, with the ecc muscle action being the one with the most significant discrepancy. Concerning hip joint AD/ABD ratio, the values fluctuate between 0.85 and 1.35, with the average value being 1.00 ± 0.14.

## Discussion

This study focused on under-19 Portuguese soccer players, studying the lower-limb knee joint differences and asymmetries. A second aim was to assess the hip abductor and adductor asymmetry. Bilateral differences and muscle asymmetries have been linked to several lower extremity injuries in soccer players [5, 6, 8]. Current study results have shown pronounced bilateral differences regarding knee joints between dominant and non-dominant legs. Slightly asymmetries were found in the knee agonist-antagonist muscles relationship and no imbalance effect on the hip joint. The present sample’s morphological and body composition characteristics are in line with the previous research samples with young soccer players [30–32]. This is of extreme importance once literature regarding strength parameters normalizes absolute values to the weight or lever length. Measurements were resulting from the isokinetic evaluation of the extension and flexion function of the knee joint in both lower limbs (i.e., 60°/s) in different muscular actions (i.e., con and ecc). The angular velocity was selected given previous studies considering soccer players [7, 30, 33]. The 60°/s angular velocity was previously used to evaluate maximum muscle resistance values [34].

Lehance et al. [9] examined the pre-season muscular strength and power profiles in professional and junior elite soccer players. This group of authors focused on con muscular action in U-21 and U-17 age categories. In confronting the current study’s values, Portuguese soccer players situated in between U-21 and U-17 Belgian players for KE (i.e., 195.5 N.m vs. 231.7 and 194.7, respectively). Considering KF, our sample presents lower strength values than Belgian peers. The KFcon / KEcon ratio considers that the measured muscular action (con, ecc, or isometric) coincides for agonist and antagonist regardless of the angular position [25]. The knee joint’s physiological movements (flexion and extension) combine eccentric muscular contraction with con muscular contraction by expressing the agonist-antagonist relationship. This KFecc / KEcon ratio tended to 1.00, representing a 1:1 relationship [17]. More recently, Eustace et al. [30] confronted 17 senior soccer professional players with 17 young U-19 elite, all of the same club of *English Football League Division 2*. The study’s purpose was to assess players bilaterally after calculating bilateral differences and considering dynamic functional ratios at specific angular positions. English players have presented ratios of 0.73 and 0.72 for a D and ND, respectively. In the current study, similar values were found (i.e., 0.77 and 0.74, respectively, for D and ND), both far from that established by international literature (1.00). This inequality maybe because of the KF deficit in ecc muscular action highlighting the importance of screening and muscular strengthening for strain injury prevention and anterior cruciate ligament (ACL) injury protection.

It is consensual that soccer players have morphological and strength asymmetries [35, 36]. These bilateral differences are justified by physical performance and movement patterns required during soccer playing, training, and training age [7], affect posture, sports performance, and increase hamstring strain rate [5]. Literature has pointed critical values with players presenting bilateral strength deficit higher than 15% being classified as asymmetrical [4]. However, young soccer players had no lower limb asymmetry in the present study since the bilateral strength deficit was lower than 15%. However, with values around 10%, the need to conduct individual evaluations to identify this potential injury risk factor should be considered in-season screening. Furthermore, in a study of 20 young Czech elite soccer players (i.e., 15.7 years), Lehnert et al. [37] also found low mean values for bilateral peak torque deficit (7% for KEcon, 4% for KFcon, and 5% for KFecc).

When assessing hip strength, the "make" technique (isometric contraction) was used instead of the "break" technique (eccentric contraction) since it presents more reliable and accurate values [38]. Furthermore, the squeeze force, being a modifiable risk factor for the occurrence of groin injuries, could be monitored in the pre-season to identify injury risk. In addition, it also can be used for monitoring during competitive periods as an injury prevention strategy [15]. However, the squeeze values of the present study (absolute and normalized to weight) are lower than the average values of Moreno-Perez et al. [29] in a cohort of 71 elite Portuguese male players (35 seniors [24.8 ± 4.2 years] and 36 U-19 players [17.4 ± 0.6 years]) who did not present groin injuries at the time of the assessment (564.0 ± 58.7 N and 7.7 ± 0.89 N/kg). The difference in chronological age can explain this discrepancy between the values presented in the present study. Moreno-Perez et al. [29] found a significantly higher adduction force production capacity once they included the senior players. Considering only the U-19 age category, Portuguese soccer players produced more squeeze force than the Australian counter peers in pre-season tests (i.e., 325–350 N) [39].

The ADD:ABD ratio is 1.00 ± 0.14, value in line with previous literature: mean of 1.08 for 27 elite Australian male soccer players (15.1 ± 0.7 years) in the pre-season assessment [40]. Monitoring the ADD:ABD ratio is essential to assess a possible difference in strength between the agonist and the antagonist, which is associated with an increased risk of injury to the adductors when the abduction strength is 20% higher than the adduction strength [41]. The season adduction development force increases the ADD:ABD ratio, improving the athlete’s condition, making the follow-up evaluation throughout the season crucial [39].

The current study demonstrates some limitations; firstly, it has been conducted on a specific sample of young male Portuguese soccer players from the same soccer academy, these findings may not be extended to other athletes’ populations. Additionally, another potential weakness is that the current information is related to the beginning of the season (ending of the pre-season, which corresponds to the academy screening program) and does not provide information on how these scores may vary during the season or from season to season. Also, we set a 12-month exclusion criterion from any previous groin/hip and knee injuries in our study sample while the strength deficit produced by a previous groin/hip injury might last more than this established period. Finally, once soccer is a sport where several dynamic actions occur, hip isokinetic strength data should be studied in future studies. Despite these limitations, the authors believe that the manuscript adds valuable information to provide an easy and helpful screening test to increase the likelihood of preventing groin injuries in young male soccer players. The current study could give reference values for young male Portuguese soccer players as practical implications. In particular, coaches and physical trainers should pay attention to eccentric Hamstring strength training to avoid lower-extremity injuries. The current study methodology may be a helpful tool to examine the dynamic knee joint stability.

## Conclusions

The main conclusions of this study are that when one-sided absolute values are compared, there are differences between the D and ND limbs in young Portuguese male soccer players. However, ipsilateral analysis with the composite measures (conventional and functional ratios) tend to be similar in both lower limbs. Strength values of the hip adductors and abductors do not show differences; functionally, there are no differences, with a balance between the agonist and antagonist muscles. In short, regardless of the strength values obtained by the isolated muscle groups, there must be a balance between the pairs of agonist-antagonist muscles.
